# Ponseti method compared to previous treatment of clubfoot in Norway. A multicenter study of 205 children followed for 8–11 years

**DOI:** 10.1007/s11832-016-0760-6

**Published:** 2016-07-19

**Authors:** Christian Sætersdal, Jonas M. Fevang, John Asle Bjørlykke, Lars B. Engesæter

**Affiliations:** 1 Department of Orthopedic Surgery, Haukeland University Hospital, 5021 Bergen, Norway; 2 Department of Pediatric Radiology, Haukeland University Hospital, 5021 Bergen, Norway; 3 Department of Clinical Medicine, University of Bergen, Bergen, Norway

**Keywords:** Clubfoot, Ponseti, Surgical treatment, Number of surgeries, Outcome, Talar flattening

## Abstract

**Purpose:**

Despite few studies comparing Ponseti treatment and traditional treatment of clubfoot (talipes equinovarus), the Ponseti method is now accepted as standard treatment for this deformity. The Ponseti method was introduced in Norway in 2003 and the purpose of this multicenter-study was to compare the results of Ponseti treatment with the results of the previous treatment for clubfoot in Norway.

**Methods:**

90 children (134 clubfeet) treated with previous treatment (pre-Ponseti group), were compared to 115 Ponseti treated children (160 clubfeet) (Ponseti group). The previous treatment consisted of casting and surgery if needed. At 8–11 years of age, all children were examined by the same orthopaedic surgeon, the parents answered a questionnaire, all feet were X-rayed and information about surgical procedures was obtained from the patient records.

**Results:**

The number of surgeries was higher in the pre-Ponseti group, and the number of extensive surgeries was 119 in the pre-Ponseti group compared to 19 in the Ponseti group. The range of motion in the ankle joint was better in the Ponseti group. Children in this group had better function, higher satisfaction and less pain according to patient and parent reported outcome measures. The incidence of moderate or severe talar flattening was higher in the pre-Ponseti group.

**Conclusion:**

Ponseti treatment seems to be superior to the previous treatment in Norway, with regards to number and severity of operations, flexibility of the foot and ankle, parent/patient reported outcome and the presence of talar flattening on X-ray.

## Introduction

Idiopathic clubfoot is a congenital deformity with multifactorial etiology in otherwise healthy children. The treatment goal is a plantigrade, flexible and pain-free foot, without deformity. Previously, a majority of the children required surgical correction. During the last two decades, the Ponseti treatment of clubfoot seems to have become the standard treatment for this deformity worldwide [1–7]. This is also concluded in a recent review article [8]. Traditionally the treatment is medical-led, but physiotherapist-led Ponseti clinics have shown equally good results, even in non-idiopathic and complex clubfeet [9].

The percentage of clubfeet treated with extensive surgery in the United States dropped from 70 % in 1996 to just over 10 % in 2006 [10]. Surprisingly few studies have, however, compared Ponseti treatment with previous treatment, and these studies are mainly single center studies with relatively few patients or short follow-up time [11–14].

Most hospitals in Norway introduced the Ponseti method in 2003, and the short-term results were good [15]. Prior to this, the treatment consisted of serial casting not according to Ponseti, followed by surgery if sufficient correction was not obtained. One study on the previous treatment found that 75 % of the feet needed extensive surgery [16]. Reported long-term complications in surgically treated clubfeet are stiffness, pain and residual deformities [3, 4, 17].

The purpose of our nation-wide multi-center study was to compare the previous treatment to Ponseti treatment with 8–11 years follow-up time. We wanted to compare possible differences between the two groups regarding (1) numbers and types of surgeries, (2) flexibility and deformity of the foot and ankle, (3) patient and parent reported pain, function and satisfaction, and (4) talar flattening on x-rays.

## Methods

### Patients

#### Pre-Ponseti group

Children born 2000–2002 with idiopathic clubfeet were scheduled for follow-up examination for this study at their clinic at the end of 2010. 90 children (134 feet) were examined. The treatment in this group differed slightly between hospitals. In general the treatment consisted of weekly changing of above-the-knee casts of either synthetic soft cast (95 feet) or Plaster of Paris (39 feet) for 13 (8–16) weeks, followed by surgery if needed. All hospitals prescribed a unilateral orthosis for approximately 18 months to prevent relapse. Indications for primary surgery or surgery due to relapse were made by the local orthopaedic surgeon based on the surgeon`s experience and the traditions at the different hospitals.

#### Ponseti group

Children born 2004–2006 with idiopathic clubfeet were scheduled for follow-up examination for this study at their clinic at the end of 2014. 115 children (160 feet) were examined. All feet were treated according to the Ponseti method with weekly changing of above-the-knee casts of either synthetic soft cast (122 feet) or Plaster of Paris (36 feet). In average 7.1 (3–13) casts were needed to correct the deformity. If needed, a tenotomy of the Achilles tendon was made [5]. This procedure was performed in the outpatient clinic in local anesthesia (7 hospitals, 102 feet), or in the operating room in general anesthesia (1 hospital, 27 feet), before the final cast was applied. A brace was used for 4 years to prevent relapse. The children used either a standard bilateral foot abduction brace (63 %), or a custom made unilateral above-the-knee brace (29 %) [15]. A majority of the children (65 %) used the brace for at least 6 h every night until 4 years of age, while 24 % of the children used the brace for more than 2 years and 11 % terminated the brace before 2 years of age. Indications for surgery due to relapse were made by the local orthopaedic surgeon based on the surgeon`s experience, the traditions at the different hospitals, and recommendations from the Ponseti group.

All patients started treatment within the first week of life, except for one delayed diagnosis (6 weeks) and one severe premature child (8 weeks), both being in the Ponseti group. Five university hospitals recruited patients to both groups, and additionally three local hospitals recruited patients to the Ponseti group only. All children with clubfeet born at these eight hospitals during this period were included in the study, and this represented a majority of the children with idiopathic clubfoot in Norway.

All children in both groups were examined at 8–11 years of age by the same pediatric orthopaedic surgeon. At this follow-up examination information about surgical procedures was obtained from the patient records, all feet were examined, the parents answered a questionnaire and the feet were x-rayed.

**Fig. 1:**
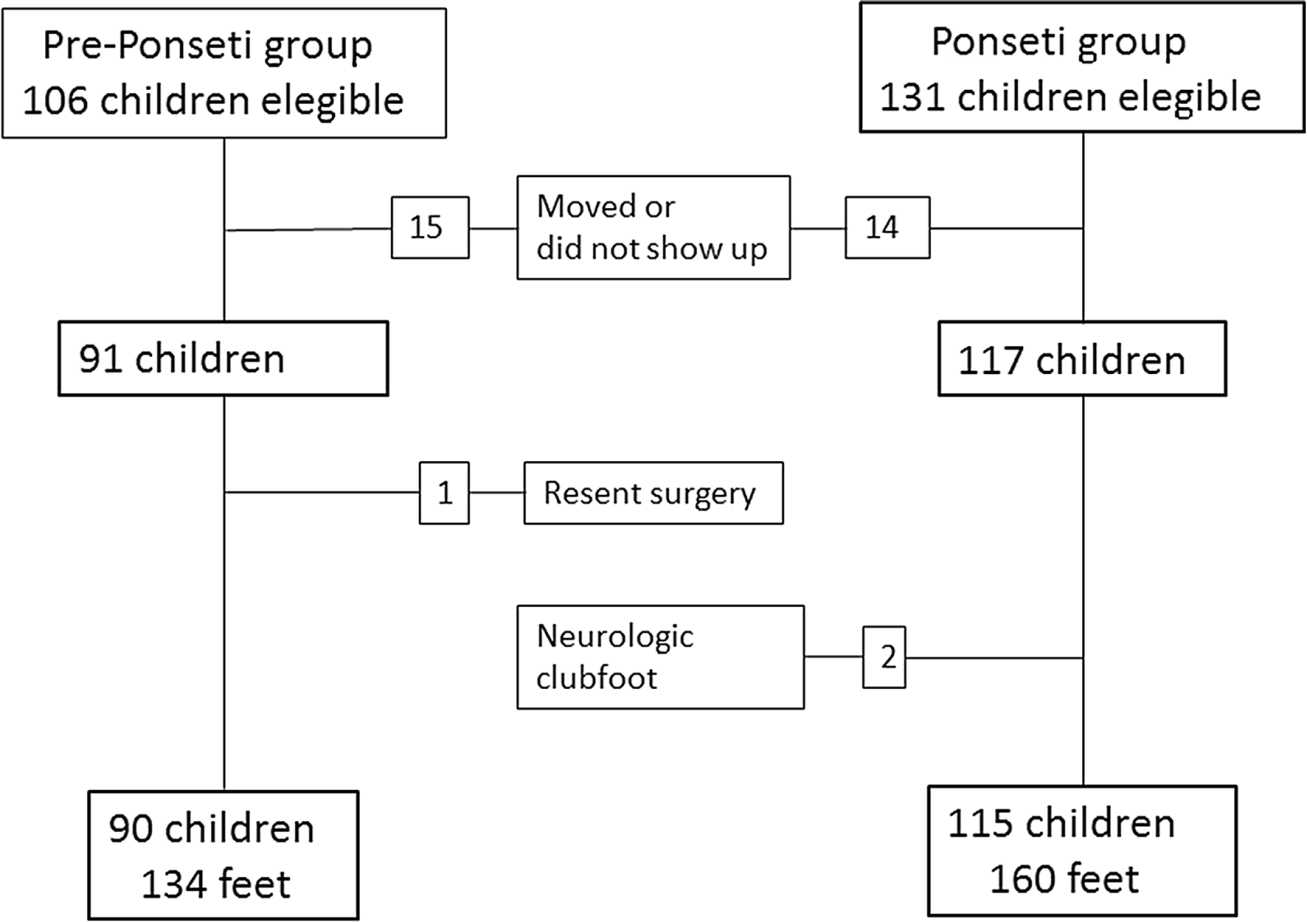
Overview of children elegible for and included in the study

Figure 1 shows the children eligible for and included in the study. Table 1 shows demographic characteristics in both groups.

**Table 1:** Patient demographics

	Pre-Ponseti group	Ponseti group
Number of children	90 (69 % boys)	115 (71 % boys)
Bilateral	49 %	39 %
Number of clubfeet	134	160
Age at follow-up (range)	9.5 years (7.9–11.0)	9.3 years (7.8–10.7)
Lost to follow-up	15	14

### Outcome measures

#### Operations

The clinical records of all patients were reviewed and operations were recorded. Tenotomy of the Achilles tendon was considered to be a part of the Ponseti method, and was not counted as an operation. Tendon lengthening, transfers and other tenotomies such as re-tenotomies, were classified as “minor surgery”. More comprehensive surgery including posterior release, posteromedial release and osteotomies were classified as “extensive surgery”. Osteotomies included Dwyer osteotomy, lengthening osteotomy of the calcaneus, and wedge osteotomies of the cuboid and the medial cuneiform.

#### Flexibility and appearance of the foot

Range of motion, intermalleolar axis and foot adduction were measured with a hand held goniometer. In children with unilateral clubfoot, we measured the following differences between the clubfoot and the normal foot: Foot length, the maximum circumference of the calf, and leg length discrepancy while standing.

#### Functional outcome

The parents answered two questionnaires regarding the child’s level of pain, function and satisfaction; the Functional Rating System for clubfoot [5], and the Disease Specific Instrument for clubfoot [18]. The Functional Rating System for clubfoot consists of three questions regarding the patient’s satisfaction (maximum 20 points), function (maximum 20 points) and pain (maximum 30 points). In addition, the examiner evaluates the foot based on position of the heel while standing (maximum 10 points), flexibility of the foot in terms of dorsal flexion in the ankle, varus-valgus movement of the heel and inversion-eversion movement of the foot (maximum 10 points all together). Finally, gait pattern is evaluated (maximum 10 points). Maximum score is 100 points, which is the best possible result. The Disease Specific Instrument for clubfoot consists of 10 questions regarding satisfaction and function, including pain. All 10 items are scaled from 1 (best) to 4 (worst). A linearly transformation is used on all items, transforming it to a 0 (worst) to 100 (best) scale [19, 20]. Each item is subsequently transformed to a 0–10 scale. The Disease Specific Instrument responses referred to the worst foot in bilateral cases. The parents answered both questionnaires together with their child.

#### Radiographic outcome

Standard anteroposterior and lateral radiographs were taken of both feet when the children met for 8–11 years follow-up examination. To compare the presence of flat top talus in the two groups, this was assessed on the lateral view, and graded from 0 (normal concentric curve) to 3 (gross flattening) [21, 22]. A consultant pediatric orthopaedic surgeon and a consultant pediatric radiologist reviewed and graded all the x-rays together. A total of 25 feet (8.5 %) were not x-rayed. Radiographic assessment could not be performed in 18 feet (6.1 %), due to poor quality of the x-rays.

### Statistical methods

SA Statistics IBM SPSS version 22.0 was used for statistical analyses. To account for bilateral observations, we analyzed the continuous data using a mixed model with a random effect to adjust for repeated measures for individuals. To compare the number of surgeries in the two groups, we used generalized estimation equations (GEE) with a Poisson distribution and a log-link, adjusted for clustered observations for individual. The radiological data were analyzed using a GEE model for binary data with a logit-link adjusted for clustered observations for individual. P-values < 0.05 were considered to be statistically significant.

## Results

### Operations

The numbers of minor surgeries, extensive surgeries and the total numbers of surgeries in the two groups are presented in Table 2. The number of extensive surgery was significantly lower in the Ponseti group. In the pre-Ponseti group, posteromedial release was the most frequent operation followed by posterior release. A total of 51 feet had more than one operation, and extensive surgery was performed more than once in 28 feet. In the Ponseti group, transfer of the tibialis anterior tendon was the most frequent operation. A second tenotomy of the Achilles tendon was performed in 12 feet, and 4 feet with were operated with a third tenotomy. In the Ponseti group, 8 feet were operated more than once.

**Table 2:** Surgical procedures

	Pre-Ponseti group *n* = 134 feet	Ponseti group *n* = 160 feet	*p* value
Minor surgey	61	43	0.01
Open tenotomies	10	16	
Open tendon lengthening	26	4	
Tibialis anterior transfer	25	23	
Extensive surgery	119	19	<0.001
Posterior release	48	12	
Postero-medial release	59	6	
Osteotomies	12	1	
Total number of operations	180	62	<0.001

Primary tenotomy of the Achilles tendon during the casting period is not included

#### Flexibility and appearance of the foot

The clinical outcomes are presented in Table 3. The dorsal and plantar flexion in the ankle joint was better in the Ponseti group. There were no differences in external rotation of the foot and ankle or foot adduction between the two groups. The intermalleolar axis/external leg torsion was reduced in the pre-Ponseti group, while the intermalleolar axis in the Ponseti group was equal to the intermalleolar axis in the healthy feet (*p* = 0.2).

**Table 3:** Clinical outcomes

	Pre-Ponseti group *n* = 134 feet (mean numbers)	Ponseti group *n* = 160 feet (mean numbers)	Mean difference (95 % conf. int.)	*p* value
Flexibility				
Dorsal flexion	16°	18°	2.3 (0.7 to 4.0)	0.005
Plantar flexion	24°	27°	2.7 (0.8 to 4.7)	0.006
External rotation	37°	37°	0.4 (−1.2 to 2.0)	0.6
Appearance				
Foot adduction	4°	4°	0.0 (−1.3 to 1.2)	0.9
Intermalleolar axis	21°	24°	2.6 (1.2 to 4.0)	<0.001
Difference, foot length	12 mm	11 mm	0.1 (−0.1 to 0.4)	0.4
Difference, leg length	3 mm	1 mm	0.2 (−0.0 to 0.3)	0.06
Difference, calf circumference	25 mm	18 mm	0.8 (0.4 to 1.2)	<0.001

Flexibility and appearance of the foot. The last 3 parameters are differences between the clubfoot and healthy foot in unilateral cases

#### Functional outcome

Children in the Ponseti group scored significantly better, both according to Laaveg and Ponseti`s Functional Rating System for clubfoot (Table 4), and according to Roye`s Disease Specific Instrument for clubfoot (Table 5). Pain as an outcome measure represented the largest difference between the two groups. Children with bilateral clubfeet in the pre-Ponseti group had significantly poorer parent/patient reported outcome than children with unilateral clubfeet, when using the Functional Rating System. The difference was also significant in the subcategories “satisfaction”, “function”, “varus-valgus flexibility” and “gait”. There was a tendency towards this difference also for the Disease Specific Instrument (*p* = 0.06). However, there were no differences in patient/parent reported outcome between children with bilateral clubfeet and children with unilateral clubfeet in the Ponseti group. We found no differences between boys and girls in parent/patient reported outcome (Functional Rating System: *p* = 0.6, Disease Specific Instrument: *p* = 0.4).

**Table 4:** Laaveg and Ponseti`s Functional Rating System for clubfoot

	Pre-Ponseti group *n* = 134 feet (mean score)	Ponseti group *n* = 160 feet (mean score)	Mean difference (95 % conf. int.)	*P* value
Parent/patient reported outcome				
Satisfaction (max. 20 pts.)	16	17	1.5 (0.7 to 2.3)	<0.001
Function (max 20 pts.)	15	17	2.1 (1.3 to 3.0)	<0.001
Pain (max 30 pts.)	22	25	2.7 (1.4 to 3.9)	<0.001
Physical examination/evaluation				
Heel position (max 10 pts.)	8.9	8.4	0.5 (–0.1 to 1.2)	0.1
Dorsal flection (max 5 pts.)	3.2	3.5	0.3 (0.0 to 0.6)	0.03
Varus-valgus (max 3 pts.)	2.6	2.7	0.1 (0.0 to 0.2)	0.03
Inversion-eversion (max 2 pts.)	2.0	2.0	0.01 (0.0 to 0.0)	0.1
Gait (max 10 pts.)	8.1	8.8	0.7 (0.3 to 1.0)	0.001
Total score (max 100 pts.)	78	84	6.9 (4.0 to 9.9)	<0.001

High score indicates good result

**Table 5:** Roye`s disease specific instrument for clubfoot

	Pre-Ponseti group *n* = 90 children (mean score)	Ponseti group *n* = 115 children (mean score)	Mean difference (95 % conf. int.)	*p* value
Satisfaction				
Status of foot	7.2	7.9	0.7 (0.1 to 1.3)	0.02
Appearance of foot	7.1	7.8	0.7 (0.1 to 1.3)	0.02
Amount of teasing	9.3	9.5	0.2 (−0.2 to 0.5)	0.2
Finding shoes that fit	6.1	7.4	1.3 (0.5 to 2.1)	0.001
Finding shoes that he/she likes	6.8	8.0	1.2 (0.4 to 1.9)	0.002
Function and pain				
Pain (no = 10, yes = 0)	2.4	5.2	2.7 (1.4 to 4.0)	<0.001
Limitations in walking	7.7	8.7	1.0 (0.4 to 1.7)	0.001
Limitations in running	6.5	7.8	1.3 (0.6 to 2.0)	<0.001
Pain during heavy exercise	6.2	7.7	1.5 (0.8 to 2.2)	<0.001
Pain during moderate exercise	7.4	8.6	1.2 (0.6 to 1.8)	<0.001
Total score (0-100 pts.)	66	79	12 (8 to 17)	<0.001

All 10 parameters are patient/parent reported outcome. All parameters are scaled from 0 (representing worst outcome) to 10 (representing best outcome)

#### Radiographic outcome

There were significantly more feet with moderate and severe talar flattening in the pre-Ponseti group (Table 6).

**Table 6:** Talar flattening on X-ray

	Pre-Ponseti group *n* = 118 feet	Ponseti group *n* = 133 feet	*P* value
Normal	10 (8 %)	16 (12 %)	
Mild	48 (41 %)	79 (59 %)	
Moderate	50 (42 %)	32 (24 %)	
Severe	10 (8 %)	6 (5 %)	0.014

16 and 27 radiological examinations were either missing or discarded due to poor quality in the two groups respectively

## Discussion

After introducing Ponseti treatment, the numbers of surgeries were considerably lower, and the surgeries were less extensive. The dorsal and plantar flexion was better in the Ponseti group, but there were no differences in external rotation and foot adduction. The parent/patient reported outcome was significantly better after Ponseti treatment. The presence and severity of talar flattening was reduced in the Ponseti group.

In the pre-Ponseti group, 81 % of the feet needed operation and 38 % of the feet needed more than one operation. This is in accordance with other studies [12, 14, 22, 23]. The material of Laaveg and Ponseti consisted of 70 patients with 104 Ponseti-treated clubfeet, ranging from 10 to 27 years. 7 of these feet were operated with posterior release or posteromedial release, and 2 were operated with triple arthrodesis. These numbers are similar to ours in the Ponseti group. A study from New Zeeland showed a 10 % rate of extensive surgery due to relapse after Ponseti treatment [12]. Other studies show lower rate of surgery due to relapse or failure after Ponseti treatment, but with short follow-up time [6, 13].

Maintaining range of motion in the ankle is one of the main objectives when treating clubfoot. Our patients had better dorsal flexion and similar plantar flexion of the ankle compared to other studies [1, 14, 17, 18, 24, 25]. The intermalleolar axis/leg torsion was reduced in the pre-Ponseti group. The normal leg torsion in the Ponseti group can be explained by the focus on extensive abduction of the foot during the casting period, and/or placing the feet in an external rotated position in the foot abduction brace.

Children in our pre-Ponseti group had a greater difference in calf circumference, and a tendency towards greater leg length discrepancy than children in the Ponseti group. Smith et al. [14] did not find these differences.

Assessing the result after clubfoot treatment can be challenging. A satisfactory physician-based result is not helpful if the child and parents are unhappy. We used two different clubfoot-specific questionnaires. The Functional Rating System for clubfoot is the most commonly used rating scheme for assessing the long term result after clubfoot treatment [3–5, 22, 26]. It was originally used by Laaveg and Ponseti on patients ranging from 10 to 27 years. In their study the average score was 87.5, compared to 84 points in our Ponseti group. Laaveg and Ponseti also made a classification according to score into excellent (90–100 points), good (80–89 points), fair (70–79 points) and poor (<70 points). Using this classification, our Ponseti group had a lower rate of excellent results (40 vs 54 %), but otherwise the results are comparable. Using the same classification in the pre-Ponseti group, our results are better than reported by Dobbs et al. [3] and inferior to the findings of Hutchins et al. [22]. A study comparing posterior release to more comprehensive release found a Functional Rating System score of 81 and 86 in the two groups [26]. These scores are better than the results in our pre-Ponseti group.

The Disease Specific Instrument for clubfoot is a parent reporting questionnaire designed for children in the same age group as in our study. It was originally used in surgically treated clubfeet [18], but the instrument was later validated for Ponseti treated clubfeet [19] and has been used in operatively treated clubfeet with longer follow-up [20, 27]. The Disease Specific Instrument score was better in the operatively treated patients in Dietz’ study (75 points), compared to both our pre-Ponseti group (66 points) and the patients in Roye`s study (68.6 points). Our Ponseti-group scored slightly poorer than Dietz’ Ponseti group (79 vs. 85.1 points). The parents answered both questionnaires, as we believe the children were too young to answer these questions alone. The children were together with their parent while the questionnaires were answered, and some of the questions were answered by the child, like the question about amount of teasing. We used only clubfoot specific questionnaires in this study. Children with idiopathic clubfeet are otherwise healthy, and general health- and quality of life questionnaires were considered less valuable. This was described by Roye et al. who in a study using the Functional Status II-R questionnaire, found that children with clubfeet scored at the top of the scale regarding general health [18]. A disease specific evaluation does also have limitations, as it reflects the parent and child’s subjective experience of the disorder which may be influenced by a number of confounders. Even so, we found the disease specific questionnaires to be most suitable for the purpose of our study.

Traditionally, radiographic measures have been used to evaluate the results of clubfoot treatment, but the relation between radiographic appearance and clinical assessment is questionable [18, 20, 28, 29]. The talocalcaneal angle is probably the best-known radiographic parameter, but this measurement varies greatly indicating that this parameter is inappropriate [18]. We wanted to investigate if there were any differences in talar flattening between the two groups. The classification of flat top talus/talar flattening was originally used in adults [21], but was later used in children and adults [22]. Dunn and Samuelson found flat top talus in all 20 feet; mild in 3/20 feet, moderate in 12/20 feet and severe in 5/20 feet. In the study of Hutchins et al., 26 % of the feet had no talar flattening, and only 1.5 % had severe flattening. The age of their patients ranged from 8 to 31 years. In terms of radiological assessment, our results are better than Dunn and Samuelsson’s, but not as good as Hutchins`.

A major strength of this study was a relative high number of patients in two comparable groups. Few patients in both groups were lost to follow-up. Interobserver variabilities were excluded by having the same person examining all children. Furthermore, this study was national and included patients and surgeons from several different hospitals, rendering the external validity of our findings high. The study was not randomized, but to our knowledge, there are no randomized studies involving Ponseti treatment. One study was originally a randomized study comparing Ponseti treatment with surgical treatment, but was converted to a prospective comparative study due to problems including children to randomization [12]. This illustrates the difficulty of conducting a randomized controlled trial on this subject. Our study, comparing two similar groups having been treated nearly at the same time and with no selection bias, is maybe the best possible study design to assess this issue. A weakness of this study could be the heterogeneity of the pre-Ponseti group, as the treatment in this group differed to some extent between the hospitals, and probably more than in the Ponseti group.

Our study was commenced immediately after introducing the Ponseti-method in Norway in 2003, and some traditions from the previous treatment was continued. This is why both soft cast and plaster of Paris was used as casting materials. Additionally, two hospitals continued to use a unilateral brace, while the rest introduced the standard bilateral foot abduction brace. This difference is a potential weakness of the study. However, the objective of the study was to compare two different treatment methods in Norway, and it may be an advantage that 7/8 hospitals used the same casting material in both groups. When introducing the Ponseti method, the bracing protocol was changed, and it was recommended to use the brace for 4 years. The importance of brace compliance in Ponseti treatment is described in a recent study [30] and a systematic review [31]. Brace compliance was good in the Ponseti group in our study.

Another challenge in clubfoot studies is indication for surgery and type of surgery if sufficient correction is not achieved during the initial treatment, or a relapse is recognized. It is very difficult to select uniform guidelines for both timing and type of surgery, and both will be depending on local hospital traditions and surgeon`s experience and preferences.

To conclude, Ponseti treatment seems to be superior to the previous treatment in Norway, with regards to number and severity of operations, flexibility of the foot and ankle, parent/patient reported outcome and the presence of talar flattening on X-ray.
